# Open letter: challenging the removal of key bacteria from the updated 2024 WHO Bacterial Priority Pathogen List

**DOI:** 10.1099/mgen.0.001475

**Published:** 2025-08-12

**Authors:** Dessislava Veltcheva, Julia Moreno-Manjón, Alejandra Dávila-Barclay, Birgitta Duim, Martin C.J. Maiden, Samuel K. Sheppard

**Affiliations:** 1 Department of Biology, University of Oxford, Oxford, UK; 2 Laboratorio 4 Edificio A4, Carrera de Médico Cirujano, Facultad de Estudios Superiores Iztacala, Universidad Nacional Autónoma de México, Mexico City, Mexico; 3 Zoonotic Disease Research Lab, One Health Unit, School of Public Health and Administration, Universidad Peruana Cayetano Heredia, Lima, Peru; 4 Department of Biomolecular Health Sciences, Faculty of Veterinary Medicine, Utrecht University, Utrecht, Netherlands; 5 Ineos Oxford Institute for Antimicrobial Research, Department of Biology, University of Oxford, Oxford, UK

**Keywords:** antimicrobial resistance (AMR), appeal, *Campylobacter*, *Helicobacter*, priority pathogens, WHO

In 2017, the World Health Organization (WHO) published the Bacterial Priority Pathogen List (BPPL) to direct research and development efforts towards the most urgent threats posed by antimicrobial resistance (AMR) bacteria [1]. The list categorized 24 bacteria–drug combinations into priority tiers using criteria such as resistance levels, disease burden and treatment availability. Since then, the BPPL has shaped AMR-related policies, funding, surveillance and public health strategy [2,8].

An updated BPPL released in 2024 [9] responds to evolving resistance and enhanced surveillance but includes concerning changes. Specifically, five clinically significant and globally prevalent pathogens were removed, including fluoroquinolone-resistant *Campylobacter jejuni* and clarithromycin-resistant *Helicobacter pylori*. Also excluded were penicillin-non-susceptible *Streptococcus pneumoniae*, third-generation cephalosporin-resistant *Providencia* spp., and vancomycin-intermediate/resistant *Staphylococcus aureus*. The report offered little explanation, only noting that decisions were based on evidence and expert consensus, despite the significant public health impact of these pathogens. Given the BPPL’s influence, such omissions without transparent justification risk undermining global efforts to address AMR.

This open letter aims to highlight concerns about the downgrading of important pathogens and argues for their reinstatement based on burden, resistance trends and the need for consistent, evidence-driven prioritization.

## BPPL inclusion criteria should be transparent, systematic and evidence-based

The 2017 WHO BPPL spurred significant investment in pathogen research. The prioritization of additional pathogens in the 2024 BPPL was welcomed and included detailed rational for prioritizing organisms including rifampicin-resistant *Mycobacterium tuberculosis* and macrolide-resistant streptococci [1]. The 2024 revision relied on 92 systematic reviews from 2017 to 2022 to assess pathogen risk [9]. Where current data were lacking, older 2017 data were reused, as with *Salmonella*, *Shigella* and *S. pneumoniae* [9]. This inconsistent approach may have unjustifiably downgraded pathogens, particularly those for which surveillance data are limited. This is often the case for organisms that are difficult to culture in resource-limited settings, including *Campylobacter* and *H. pylori*. In fact, it could be argued that data scarcity should prompt better monitoring, not de-prioritization.

There was little or no justification for de-prioritizing organisms such as *Campylobacter* and *H. pylori* that remain globally abundant. This falsely implies declining disease prevalence and/or resistance, despite evidence to the contrary. For example, fluoroquinolone resistance continues to rise in *Campylobacter* [10]. The downgrading of pathogens without adequate explanation risks halting momentum of research and control efforts. It is important that the criteria for removal from the WHO BPPL are made publicly available. Without this, stakeholders may misinterpret downgrading as evidence that a pathogen is no longer a threat, which could lead to reduced research, surveillance and stewardship activities.

## Pathogen prioritization supports effective intervention

Fluoroquinolone-resistant *Campylobacter* and clarithromycin-resistant *H. pylori* are globally important pathogens that exemplify the growing AMR crisis. *C. jejuni* is one of the most common causes of foodborne diarrhoeal disease world-wide and is linked to serious sequelae [11]. In low- and middle-income countries (LMICs), repeated infections lead to a cycle of chronic diarrhoea linked to malnutrition and stunting [12]. Treatment failure is associated with fluoroquinolone resistance, rising in the UK from 5% in 1998 to 45% in 2018 [10], with 48% and 90% resistance recently reported in Chile and Peru, respectively [13,15]. Equally important is the stomach-dwelling pathogen *H. pylori* that infects over half the world’s population, causing gastritis, peptic ulcers and gastric cancer, especially in LMICs [16,17]. Clarithromycin resistance has rendered standard *H. pylori* triple therapy ineffective, forcing reliance on second-line regimens. Resistance prevalence has been estimated at 30–50% in South America, the Middle East and China [18,20] with rising trends in Australia and Europe [21,22]. Given its role in gastric cancer, one of the leading causes of cancer deaths in LMICs [23], the downgrading of *H. pylori* appears difficult to justify.

Macrolides are often the last effective treatment for *Campylobacter*, but they are not always accessible or affordable. In *H. pylori*, macrolide resistance has rendered first-line therapy obsolete in many regions, necessitating more expensive and less accessible regimens. Inappropriate antibiotic use, weak stewardship and global travel contribute to the spread of resistance with few plans for new therapies [1]. Including these bacteria on the BPPL supported drug and vaccine research, improved surveillance and stewardship, and raised awareness among clinicians and policymakers. Their removal from the BPPL could reduce investment in diagnostics, treatment and surveillance despite urgent need.

## Recommendations

Authoritative organizations such as the WHO have a major leadership role in confronting the existential threat posed by rising AMR [24,25]. For this reason, and to preserve the BPPL’s utility, we propose that the WHO systematize an approach for pathogen inclusion, based upon transparent criteria of AMR pathogen importance. Under such a system, it is highly likely that previously listed pathogens, including fluoroquinolone-resistant *C. jejuni* and clarithromycin-resistant *H. pylori*, would be reinstated in the BPPL. This is extremely important as prioritization affects global research priorities, surveillance and funding decisions [2,2, 3, 7, 8, 26]. Diarrhoeal and respiratory pathogens remain leading causes of death in children under five in LMICs [27]. Although adding rifampicin-resistant *M. tuberculosis* is a welcome step, removing *Campylobacter* undermines efforts to address AMR in paediatric diarrhoea. While the 2024 BPPL improved transparency by using 92 systematic reviews [9], limited LMIC surveillance, especially for *Campylobacter*, likely led to the underestimation of the burden. This should not imply reduced threat, but a call for better monitoring. We urge the WHO to apply consistent, evidence-based criteria and to adopt a country-based approach that reflects regional AMR trends.

## Abbreviations

AMR: Antimicrobial Resistance
BPPL: Bacterial Priority Pathogen List
C.jejuni: Campylobacter jejuni
H.pyrori: Helicobacter pylori
LMIC: Low and Medium-Income Countries
M.tuberculosis: Mycobacterium tuberculosis
S. pneumoniae: Streptococcus pneumoniae
WHO: World Health Organisation
